# Short-lasting unilateral neuralgiform headache with conjunctival injection and tearing (SUNCT) with preserved refractory period: report of three cases

**DOI:** 10.1007/s10194-011-0412-7

**Published:** 2012-01-07

**Authors:** Vimal Kumar Paliwal, Prabhat Singh, Achal Kumar, Sushil Kumar Rahi, Rakesh Kumar Gupta

**Affiliations:** 1 Department of Neurology, Sanjay Gandhi Postgraduate Institute of Medical Sciences, Lucknow, 226014 Uttar Pradesh India; 2 Department of Radiology, Sanjay Gandhi Postgraduate Institute of Medical Sciences, Lucknow, 226014 Uttar Pradesh India

**Keywords:** Short-lasting unilateral neuralgiform headache with conjunctival injection and tearing (SUNCT), Refractory period, Trigeminal neuralgia with autonomic features, Short-lasting unilateral neuralgiform attacks with cranial autonomic features (SUNA)

## Abstract

**Background:**

Short-lasting unilateral neuralgiform headache with conjunctival injection and tearing (SUNCT) and short-lasting unilateral neuralgiform attacks with cranial autonomic features (SUNA) are rare primary headache syndromes characterized by spontaneous or triggered attacks of unilateral, brief, multiple, orbitofrontal pain associated with ipsilateral autonomic features. SUNCT is considered as a subset of SUNA. In SUNA, there may be cranial autonomic symptoms other than conjunctival injection and lacrimation, or either of two is present. SUNCT/SUNA can be triggered immediately after or at the decrescendo phase of the ongoing attack without any intervening refractory period. Refractory period is usually present in trigeminal neuralgia. Absent refractory period is thought to reliably differentiate SUNCT/SUNA from trigeminal neuralgia and has been proposed for inclusion into the International Classification of Headache Disorders (ICHD) diagnostic criteria for SUNCT.

**Case reports:**

We report three patients of SUNCT syndrome with preserved intervening refractory period of variable duration observed at different times.

**Discussion:**

Trigeminal neuralgias with autonomic features, SUNA and SUNCT share a common pathophysiological mechanism and actually represent a continuum. It is well known that patient with trigeminal neuralgia may transform into SUNCT/SUNA. Similarly, being a continuum, the presence or the absence of refractory period and its duration may change in a patient with SUNCT/SUNA at different time points.

**Conclusion:**

The presence of refractory period should not exclude the diagnosis of SUNCT in a patient with other clinical features suggestive of SUNCT.

**Electronic supplementary material:**

The online version of this article (doi:10.1007/s10194-011-0412-7) contains supplementary material, which is available to authorized users.

## Introduction

Short-lasting unilateral neuralgiform headache with conjunctival injection and tearing (SUNCT) is a rare primary headache characterized by short-lasting unilateral orbitotemporal neuralgiform attacks with conjunctival injection, tearing and rhinorrhoea. Attacks are usually severe, brief and vary in duration from 5 to 240 s and in frequency from 1/day to 30/hour [1]. Unlike trigeminal neuralgia, SUNCT patients can trigger headache without an intervening refractory period [2]. Absent refractory period has been proposed as mandatory criteria for diagnosing SUNCT. We report three cases of SUNCT syndrome with preserved refractory period. All three cases had superior cerebellar artery loop around the root entry zone of ipsilateral trigeminal nerve.

### Case 1

**Fig. 1:**
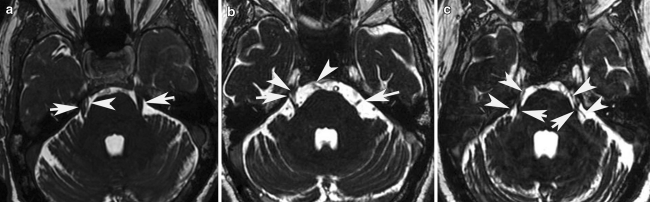
Axial MRI fast imaging employing steady-state acquisition (FIESTA) sequence showing superior cerebellar artery loop (*white arrow heads*) at root entry zone of trigeminal nerve (*white arrows*) on right side (**a**, **b**) and on both sides (**c**)

A 62-year gentleman complained of recurrent brief (20–30 s) episodes of right periorbital pain for last 2 years. Starting with 1–5 episodes/day, his pain attacks increased to 20–50 attacks/day for last 2 months. In between attacks, he remained asymptomatic. Pain was severe, non-throbbing, stabbing and most episodes were accompanied by lacrimation and redness of right eye. Pain produced visible distress but without motor restlessness (supplementary video). There was no photophobia, phonophobia, vomiting or exacerbation of pain with head movement. Pain could be triggered by touching right periorbital area and mouth opening. Patient reported absent refractory period. There was no family history of migraine. NSAIDS did not relieve his pain. Examination of the patient was unremarkable. A triggered attack with conjunctival congestion and lacrimation was witnessed (supplementary video). Refractory period of variable duration (1–10 min) was observed at different occasions. MRI of the head revealed vascular loop around right trigeminal nerve (Fig. 1a). A trial of indomethacin and oxygen inhalation failed to produce any response. Oxcarbazepine (900 mg/day) reduced the frequency of headache attacks. Oxcarbazepine induced sedation and ataxia precluded further increase in its dose. Lamotrigine was added (200 mg/day). It produced remission for last 6 months.

### Case 2

A 56-year gentleman complained of intermittent, brief (10–90 s) attacks of severe right supra-orbital lancinating pain attacks for 1 year. Episodes of attacks increased to 25–30/day from 1 week from initial 1–6/day. Inter-attack period was asymptomatic. Lacrimation, mild redness of right eye and rhinorrhoea (with longer attacks) accompanied most attacks. Pain could be triggered by touching right forehead, spitting and chewing. There was no photophobia, phonophobia, vomiting, associated motor restlessness or pain exacerbation with head movement. There was no history of refractory period. There was no family history of migraine. Carbamazepine, topiramate, amitriptyline, gabapentin and pregabalin received in last 1 year did not work. Neurological examination was normal. A triggered attack conforming to history was witnessed. An intervening refractory period of 10 min or more was observed in most witnessed episodes. MRI of the head showed superior cerebellar artery loop around right trigeminal nerve (Fig. 1b). A trial of indomethacin and oxygen inhalation did not relieve his pain. With lamotrigine (200 mg/day) he achieved remission for last 1 year.

### Case 3

A 64-year gentleman presented with recurrent, brief (10–30 s) attacks of severe, stabbing pain involving right periorbital area for 6 months. For 1 month, his attacks increased to 5–30/day from initial 1–2/day. Most episodes were accompanied by lacrimation, redness and partial drooping of right eye lid (with longer attacks). There was no photophobia, phonophobia, vomiting or restlessness. Touching right eyelid and teeth brushing could trigger attack. Patient was not sure about the refractory period. There was no family history of migraine. At the time of consultation, he was taking carbamazepine (600 mg/day) for a week with resultant 2–3 attacks/day. His clinical examination was normal. One spontaneous attack (25 s long) with conjunctival congestion and lacrimation was witnessed. Repeated attempts to trigger the headache failed; however, few spontaneous attacks were witnessed upon drug discontinuation. His MRI of the head showed superior cerebellar artery loop around both trigeminal nerves (Fig. 1c). He was restarted on carbamazepine (400 mg/day) which promptly abolished his spontaneous attacks.

## Discussion

Clinical features of our cases conform to diagnostic criteria of SUNCT (ICHD 2nd ed) and does not comply with diagnosis of migraine, paroxysmal hemicrania and cluster headache [3]. However, these attacks are similar to “trigeminal neuralgia with autonomic symptoms” which is indistinguishable from short-lasting unilateral neuralgiform headache with cranial autonomic features (SUNA) [4–6]. Unlike SUNCT/SUNA, refractory period is usually present in trigeminal neuralgia. Absent refractory period is thought to reliably differentiate SUNCT/SUNA from trigeminal neuralgia and has been proposed as mandatory criteria for diagnosis of SUNCT [2]. Our patients 1 and 2 could not demonstrate absent refractory period despite giving history of same. Owing to overwhelming pain with every trigger, they possibly presumed that a trigger just at termination of ongoing attack will also induce pain. Hence, history of refractory period (as derived from telephonic interview) needs objective verification.

Trigeminal autonomic reflex with central disinhibition of variable extent is thought to produce a continuum of trigeminal neuralgia, SUNA and SUNCT [1]. Most patients with these headaches show variation in pain intensity, attack frequency and severity of autonomic symptoms. The variations may be good enough to switch a patient from trigeminal neuralgia to SUNA/SUNCT [7–9]. Variations in severity of autonomic features and duration of refractory period were observed in all our patients. We propose that at the extreme of the continuum represented by SUNCT, little variation in the number, length and severity of pain attacks and length of refractory period (or its absence) may be seen.

All our patients had aberrant vascular loops around ipsilateral trigeminal nerve. Secondary SUNCT may have atypical features, but refractory period in these cases was not studied [7–9]. With paucity of literature, it is difficult to speculate that refractory period could be a more frequent feature of secondary SUNCT.

SUNCT caused by ipsilateral superior cerebellar artery vascular loop near the root entry zone of trigeminal nerve is rare [10, 11]. These aberrant vessels can be delineated by special MRI techniques like fast imaging employing steady-state acquisition (FIESTA). Once identified, vascular decompression over trigeminal nerve may be offered in those with failed medical treatment [11].

We conclude that a brief refractory period may exist in SUNCT patients. Refractory period may be prospectively studied in a reasonable cohort before its application as criteria for diagnosing SUNCT.

## Electronic supplementary material

Below is the link to the electronic supplementary material.

Supplementary material 1 (WMV 4223 kb)
